# Out-of-hours care in western countries: assessment of different organizational models

**DOI:** 10.1186/1472-6963-9-105

**Published:** 2009-06-23

**Authors:** Linda Huibers, Paul Giesen, Michel Wensing, Richard Grol

**Affiliations:** 1 Scientific Institute for Quality of Healthcare, Radboud University Nijmegen Medical Centre, PO box 9101, 6500 HB Nijmegen, The Netherlands

## Abstract

**Background:**

Internationally, different organizational models are used for providing out-of-hours care. The aim of this study was to assess prevailing models in order to identify their potential strengths and weaknesses.

**Methods:**

An international web-based survey was done in 2007 in a sample of purposefully selected key informants from 25 western countries. The questions concerned prevailing organizational models for out-of-hours care, the most dominant model in each country, perceived weaknesses, and national plans for changes in out-of-hours care.

**Results:**

A total of 71 key informants from 25 countries provided answers. In most countries several different models existed alongside each other. The Accident and Emergency department was the organizational model most frequently used. Perceived weaknesses of this model concerned the coordination and continuity of care, its efficiency and accessibility. In about a third of the countries, the rota group was the most dominant organizational model for out-of-hours care. A perceived weakness of this model was lowered job satisfaction of physicians. The GP cooperative existed in a majority of the participating countries; no weaknesses were mentioned with respect to this model. Most of the countries had plans to change the out-of-hours care, mainly toward large scale organizations.

**Conclusion:**

GP cooperatives combine size of scale advantages with organizational features of strong primary care, such as high accessibility, continuity and coordination of care. While specific patients require other organizational models, the co-existence of different organizational models for out-of-hours care in a country may be less efficient for health systems.

## Background

Appropriate out-of-hours care is important for a well-functioning health care system. Health policy makers all over the world are concerned about the accessibility, quality, and efficiency of out-of-hours care. In many countries the organization of out-of-hours care has changed in recent years [1-4]. Reasons for these changes include high physicians' workload, shortage of physicians and desire for separation of work and private life among physicians [3-8]. Nevertheless, other problems have remained unresolved, such as the number of self-referrals with non-urgent problems, fragmentation of out-of-hours care, unclarity for patients regarding choice of provider [9-11]. A systematic analysis of prevailing organizational models for out-of-hours care was done to identify their potential strengths and weaknesses.

Based on literature, we identified nine organizational models for out-of-hours care, which are currently used across the world (Table 1). The individual general family practice, rota groups and accident and emergency (A&E) departments of hospitals are well known and have often been described. The literature also described some relatively new models, for example the primary out-of-hours care integrated in the hospital, deputizing services, minor injury centers and walk-in-centers [5][12-14]. These models are frequently family doctor based, in small and large scale organizations. Little information was available regarding other recent developments, such as new models. Research on primary care out-of-hours models in Denmark, the Netherlands and the United Kingdom seemed to have a positive outcome, but this mainly concerned general practice (GP) cooperatives [4,13].

**Table 1:** Organizational models for out-of-hours care

**Organizational model**	**Definition**	**Example**
*Small family doctor based models (registration at a family doctor practice)*		

Individual general family practice	The GP takes care of his own patients 24 hours a day, 7 days a week.	Rural areas of Austria

Rota groups (rota)	GPs who are active in the same region take turns being on duty out-of-hours for the patient population of all (up to 15) members of the rota group	Municipalities in Norway

*Large family doctor based models (independent of registration at a family doctor practice)*		

GP cooperatives	GPs work in a non-profit organization and take turns being on duty out-of-hours for the patient population of all participating GPs. These are large-scale organizations that are supported by nurses, management, chauffeurs, et cetera.	Mostly used model for out-of-hours primary care in the Netherlands

Primary care centers (PCC)	Centers, which patients can visit without an appointment for minor injuries or illnesses. Such centers operate under supervision of a general practitioner or family physician.	In Slovenia one PCC (of all daytime centers) functions as out-of-hours center

Deputizing services	Commercial agencies that employ GPs to take over duties of other GPs.	NHS direct is common in the United Kingdom

Minor injury centers or walk-in-centers	Centers, which patients can visit without an appointment for minor injuries or illnesses in order to ask a trained nurse for health information, advice and treatment.	Ireland has a few privately organized models

*Hospital based and national models*		

Telephone triage and advice services (TTA)	Patients have contact with a medically trained professional via a fixed, non-regional, telephone number. This person advises or refers the patient to the most suitable professional.	National call center in Portugal

Emergency departments of hospitals (A&E)	Emergency departments of hospitals taking care of patients out-of-hours.	Unofficially used by patients in Belgium

Primary out-of-hours care integrated in the hospital	Primary out-of-hours care integrated in the hospital (for example, in emergency departments).	Some experiments in Italy

Critical evaluation of currently existing models should provide further insight in their performance. Information about the strengths and weaknesses of these models is needed for evidence based health care policy. The aim of our study was to assess the prevailing organizational models for out-of-hours care in order to identify their potential strengths and weaknesses.

## Methods

### Design

We performed an international web-based survey in a sample of key informants in 2007.

### Sample

The sample of key informants was purposefully selected. Key informants in national health organizations in western countries were approached, using the European Association for Quality in General Practice/Family Medicine (EQuiP) and the World Association of Family Doctors (Wonca). They were directly involved in making health policy and therefore have extensive knowledge of the organization of out-of-hours primary care in their country. We excluded delegates from countries without a health care system according to western standards [15], and some very small countries.

Firstly, an e-mail with an announcement of the study was sent to 48 key informants. They were requested to provide names and e-mail addresses of other professionals with expert knowledge within the field (snowball sampling). An e-mail containing a link to the online questionnaire with a unique invitation code was sent 1 week later. In the questionnaire, respondents were asked again to provide contact information for additional key informants within the same country. Due to this snowball sampling more and more e-mail addresses were collected. Finally, 84 extra individuals were included in the professional sample, giving a total of 145 individuals. The informants were mostly GP's, some of whom partly worked at a university or health care organization. After 1 and 2 weeks reminders were sent to increase the response rate.

### Measures

A draft questionnaire was created and after consultation of experts with much experience in international health care, a final version was provided. The paper questionnaire was transformed to an English online version. The main measures were nine organizational models for out-of-hours care, which we had identified (Table 1), the dominant model in a country (if any), and perceived problems regarding this model. For the dominant model, informants rated eight different aspects about perceived problems; continuity of care, efficiency of the model, accessibility, coordination of care, satisfaction of physicians and other professionals, patient satisfaction and safety of triage. These aspects were rated on a five-point Likert scale (no, few, some, many and major). Finally, national plans for changes in the organization of out-of-hours care in the near future and the rationale behind them were listed.

After the first analysis it was evident that informants mentioned different models within the same country and, in some cases, used other definitions of out-of-hours care organizational models. Therefore, controlling and clarifying questions were sent by e-mail to the individual respondents to make sure that the interpretation of the data was correct. We presented them the answers of other informants from their country. Information from these additional questions was processed and data was corrected where necessary.

### Data-analysis

Descriptive frequencies were used to determine the number of countries in which the nine models were used. Regarding the perceived problems of the dominant model, many or major problems were interpreted as a potential weakness. No or few problems resulted in potential strengths. Because of this recoding we made the assessment more explicit. Furthermore, we divided the organizational models into small family doctor based models (individual family doctor and rota group), large family doctor based models, and hospital based and national models to compare their performance. We did not perform statistical tests because our informants did not comprise a random sample.

## Results

A total of 71 individuals completed the questionnaire (response rate of 50%). A total of 25 countries were represented in this sample (Table 2). From Finland and Hungary we did not receive response.

**Table 2:** Participants

**Country**	**#**
Australia	2
Austria	3
Belgium	7
Canada	2
Croatia	1
Czech Republic	2
Denmark	1
France	3
Germany	1
Greece	5
Iceland	2
Ireland	1
Israel	1
Italy	4
The Netherlands	2
New Zealand	2
Norway	6
Poland	3
Portugal	1
Slovenia	6
Spain	1
Sweden	5
Switzerland	4
United Kingdom	4
United States of America	2

**Total**	**71**

### Description of models

In many countries different organizational models for out-of-hours care existed alongside each other, varying from three to nine models (Table 3). In Australia, Canada, Ireland, New Zealand, the United Kingdom and the United States of America as well as in Norway and Belgium all nine organizational models were used.

**Table 3:** Overview of countries, dominant model for out-of-hours care, and planned changes

**Country**	**Respondents (N)**	**Models (N)**	Dominant model*	**Planned changes**
Croatia	1	3	Emergency department	-
Czech Republic	2	3	Primary care integrated in hospital	Upscale care, patient fee, integrate GP coop and A&E department
Denmark	1	4	Telephone triage and advice service	Upscale care
Israel	1	4	Emergency department	-
Portugal	1	4	Primary care center	-
The Netherlands	2	4	GP cooperative	Upscale care, integrate CP coop and A&E department
Germany	1	5	Rota group	-
Iceland	2	5	Primary care center GP cooperative	-
Slovenia	6	6	Rota group	Change organization, upscale care
Spain	1	6	Telephone triage and advice service	Upscale care
Austria	3	7	Rota group	Upscale care, change structure
Greece	5	7	Individual general family practice	Upscale care, change organization
Poland	3	7	-	Change organization
France	3	8	Emergency department Rota group	Upscale care
Sweden	5	8	GP cooperative	Centralization of out-of-hours calls and triage, change organization
Switzerland	4	8	Rota group	Upscale care, call center service
Belgium	7	9	Rota group	Upscale care, centralization of out-of-hours calls and triage
Canada	2	9	Emergency department	Upscale care
Italy	4	9	Other (Guardia Medica)	Upscale care
New Zealand	2	9	GP cooperative Rota group	-
Australia	2	10	Individual general family practice GP cooperative	Improve access to high quality health care services
Ireland	1	10	GP cooperative	Upscale care
Norway	6	10	Rota group	Upscale care, enhance uniformity
United Kingdom	4	10	Deputizing service	-
United States of America	2	10	Rota group	Many different approaches

*Dominant model is the model mentioned by the majority of respondents from one country (> 50%).

According to the informants, the A&E department existed in all countries. Only in the Czech Republic and Denmark it was not used for out-of-hours primary. The primary care center (PCC), primary care integrated in the hospital, GP cooperative and rota were also present in many countries. Informants from nine countries qualified the rota group as the dominant organizational model for out-of-hours care in their country (Table 3). The GP cooperative was mentioned frequently, as was the A&E department. The PCC and the telephone triage and advice service were the dominant organizational model in five and four countries respectively. Rarely mentioned were the individual general family practice, deputizing service, minor injury unit, primary care integrated in the hospital and 'other'. 'Other' referred to the Guardia Medica, a unique out-of-hours care model in Italy.

### Strengths and weaknesses of different models

In order to compare the organizational models for out-of-hours care, we assumed that they were reasonably identical in the different countries. This assumption was confirmed by the descriptions of the models by informants.

Concerning the A&E department, satisfaction of patients with the model was a strength (Table 4). However, weaknesses of the A&E department concerned continuity of care, efficiency, coordination of care and accessibility. Overcrowding of A&E departments was mentioned frequently, by respondents from seven countries. Note that in three countries the A&E department was the only dominant model, whereas in 4 countries the rota group and the GP cooperative coexisted as dominant models. The rota group and GP cooperative seem to have complementary strengths.

**Table 4:** Perceived strengths and weaknesses of different models

	**Small family doctor based models**		**Large family doctor based models**			**Hospital based and national models**		
	
	**Individual general family practice** (N = 3)	**Rota group** (N = 21)	**GP coopera-tive** (N = 9)	**Primary care center** (N = 5)	**Deputizing service** (N = 3)	**A&E department** (N = 7)	**Telephone triage and advice** (N = 3)	**Integrated care** (N = 1)
Continuity of care	-	0	0	-	-	-	-	+
Efficiency	0	0	+	-	-	-	0	+
Accessibility	+	+	+	+	0	-	+	0
Coordination of care	0	0	+	-	-	-	0	+
Satisfaction physicians	0	-	+	-	0	0	-	0
Satisfaction other professionals	0	0	+	0	+	0	-	0
Satisfaction patients	0	+	+	0	0	+	+	-
Safety of triage	0	+	+	0	0	0	0	+

***Legend ***+ = potential strength, no or few problems (median < 2); 0 = neutral, some problems (median = 3); - = potential weakness, many to major problems (median > 2). Changes after the second mailing led to some missings; therefore, the number of most used models is lower.

The rota group of family doctors had several perceived strengths, such as accessibility, satisfaction of patients and safety of triage. The main weakness of the rota group was lowered job satisfaction of physicians. The individual general family practice was considered neutral for most aspects (strength neither weakness), but poor continuity of care was considered a weakness and high accessibility a strength. Informants from four countries mentioned a perceived lack of willingness of family doctors to participate in out-of-hours care. Furthermore, shortage of family doctors, particularly in rural areas, were mentioned by informants of four countries.

Informants who indicated the GP cooperative was the dominant organizational model in their country, mentioned many strengths, concerning for example coordination of care, accessibility and efficiency of healthcare delivery. No weaknesses were mentioned by the informants. In the eight countries where the GP cooperative was a dominant model, out-of-hours care was also provided by the rota group and the PCC as dominant models.

The PCC had high accessibility as a strength. However, continuity of care, efficiency, coordination of care and the satisfaction of physicians were weaknesses. PCC was a dominant model in four countries, but shared this position with other models such as the GP cooperative in three countries. Telephone triage and advice service had a few strengths, accessibility and satisfaction of patients. Additional dominant models in three out of the four countries had complementary strengths.

In general, continuity of care was seen as a weakness of all models, except for the integrated care model. Also, lowered satisfaction of physicians was a weak aspect of many models. Safety of triage was rated moderately or good for all models. Lowered satisfaction of patients was mentioned as a weakness of the integrated care model and poor accessibility as a weakness of the A&E department (Table 4).

### Types of organizational models

We divided the organizational models into small family doctor based models (individual general family practice and rota group), large family doctor based models, and hospital based and national models (Table 1). Our informants reported that small family doctor based models performed well. Accessibility was a strength, and also satisfaction of patients and safety of triage were assessed relatively positive. On the other hand, satisfaction of physicians was perceived a weakness, as was continuity of care. Interestingly, large scale family doctor based models (GP cooperative, PCC and deputizing services) seemed to perform even better, especially the GP cooperative. They were evaluated more positively regarding satisfaction of physicians and other professionals. Noticeable was the relatively negative assessment of the PCC; only the accessibility was a strength. Informants reported that hospital based and national models performed moderately. In general, these models had several weaknesses and few strengths (Table 4).

### Planned changes

Most of the countries had plans to change the out-of-hours care in the future, mainly changes toward large scaled organizations, integration of primary care with A&E departments and introduction of one national telephone number with centralization of out-of-hours calls and triage (Table 3). Respondents frequently indicated that reduction of fragmentation of out-of-hours care is necessary. The major reasons for changes mentioned were work dissatisfaction among family doctors, shortage of family doctors and lack of motivated family doctors for out-of-hours care. Other reasons were the overcrowding of A&E departments by primary care patients (so called self-referrals), reduction of costs and improving safety, quality and continuity of care.

## Discussion

This international survey showed that up to nine different organizational models for out-of-hours care are currently used in western countries, often different models alongside each other. The A&E department, which exists in almost all countries, was perceived to be associated with many weaknesses. Patient satisfaction was the only strength mentioned. The rota group exists in a considerable number of countries. It had many strengths, according to our informants, but it was associated with lowered job satisfaction among physicians [2,3]. GP cooperatives were perceived to have many strengths, but reduced continuity of care was mentioned as a possible weakness. Interestingly, the only perceived strength of PCC was good accessibility. Furthermore, the performance of the integrated care model seemed positive, but more information is needed to evaluate this specific organizational model. Overall, suboptimal continuity of care is considered a weakness in all organizational models except for the integrated care model.

The informants assessed the GP cooperatives most positively. The underlying factor might be that this organizational model combines size of scale advantages with characteristics of strong primary care, such as high accessibility, continuity and coordination of care [16]. The A&E department is expected to have size of scale advantages as well, but it was perceived to have weak efficiency, coordination of care and accessibility. These weaknesses probably have a relation with the overcrowding by self-referrals as a result of the unlimited access, and unnecessary resource use [9], which probably reduce the size of scale advantages. Primary care health centers are used both during daytime and out-of-hours, and in a region one health center is often used as the out-of-hours center. This extended use of these centers may account for the less positive assessment compared to the GP cooperative.

Safety of triage was a strength of all organizational models according to the informants. This is remarkable since recent research is less positive regarding safety of telephone triage at GP cooperatives [17-19] and appropriate referral rates at call centers [20]. These results indicate that triage is not optimal and suggests that further research is needed that emphasizes service use, safety, cost and patient satisfaction [21].

Continuity of care was often considered a weakness, even for the individual family doctor practice. Continuity is not just one family doctor who treats his own patients (personal continuity), but also continuity through the entire health care system (information continuity and treatment continuity). Only the integrated care model was perceived to have a high level of continuity of care, perhaps because of the collaboration and teamwork, which also has a positive impact on coordination of care. On the other hand, lowered satisfaction of patients was mentioned as a weakness of integrated care models. This might be the result of the perception that patients will be less satisfied if they consult a family doctor instead of a specialist and do not receive diagnostics. Efficiency is considered a strength of both the GP cooperative as the integrated care model. The underlying mechanisms are obviously avoidance of overtreatment (e.g. advice by telephone instead of face-to-face contact, if possible) as well as avoidance of undertreatment (e.g. adequate recognition of and action on highly urgent health problems). In other words, efficiency reflects an optimal relation between resource use and effectiveness (not just reduced costs). Large-scale GP cooperatives may improve satisfaction of GPs by reduction of workload and more pleasant frequency of shifts so that professional work and private life can be combined [1-7][8].

Our study had some limitations. A purposeful sample was used, by selecting known contacts from existing organizations for primary care. This might lead to selection bias, in particular regarding the perspective on primary care. Most of the informants that participated in this study had a GP perspective. Definitions of the organizational models were based on the literature. The apparent use of various definitions made individual comparison less reliable and by grouping the comparable models we corrected for interpretation differences. Also, we achieved a more compact and general view of performance. In many countries the out-of-hours care is fragmented and many organizational models exist alongside. Consequently, respondents have their own regional perspective and individual knowledge. Therefore, a second mailing was conducted to achieve a greater correlation between respondents from the same country. The final results have been double-checked to increase reliability. Furthermore, we recoded the assessment of the criteria into three categories, to make the assessment more explicit. It is important to realize that we presented merely a semi quantitative overview.

Our overview provides an impression regarding the models and shows a trend in out-of-hours care organization. GP cooperatives stand out and are mentioned in future plans, as is integrated care. The aim in the future should be to evaluate the models empirically, focusing on large scale models and integrated care. Therefore, research is needed on the quality of different models. Furthermore, national health care systems influence the feasibility of an organizational model, despite the assessment of different models. Therefore, investigating regional motives to choose for an organizational model for out-of-hours care, such as local geography and community authorities, is needed as well. It would also be interesting to repeat this study in order to investigate the changes, possibly with a focus on national health care systems.

## Conclusion

In conclusion, large scale family doctor based organizational models and integrated care models for out-of-hours care seemed to have many strengths. This finding should inform decision makers in healthcare. Furthermore, continuity in out-of-hours care needs attention regardless of the models. Not surprisingly, the planned changes in the near future are aimed to address these problems. The plans comprise of further development towards large-scale organizational models and integrating care models. Reasons for changes are uniform in the different countries and seem to be related to the performance.

## Competing interests

The authors declare that they have no competing interests.

## Authors' contributions

LH, the main investigator, developed the instrument, performed the data collection and the analysis and drafted the manuscript. PG contributed to the design, development of the survey and helped to draft the manuscript. MW has participated in discussions about the design of the study, the instrument and helped to draft the manuscript. RG is involved in the design of the study, the analyses and helped to draft the manuscript. All authors have read and approved the final version of the manuscript.

## Pre-publication history

The pre-publication history for this paper can be accessed here:



## References

[B1] Christensen MB, Olesen F (1998). Out of hours service in Denmark: evaluation five years after reform. BMJ.

[B2] Giesen P (2007). Quality of out-of-hours primary care in the Netherlands. PhD thesis.

[B3] van Uden CJ, Giesen PH, Metsemakers JF, Grol RP (2006). Development of out-of-hours primary care by general practitioners (GPs) in The Netherlands: from small-call rotations to large-scale GP cooperatives. Fam Med.

[B4] Grol R, Giesen P, van Uden C (2006). After-hours care in the United Kingdom, Denmark, and the Netherlands: new models. Health Aff (Millwood).

[B5] Hallam L (1994). Primary medical care outside normal working hours: review of published work. BMJ.

[B6] Hansen BL, Munck A (1998). Out-of-hours service in Denmark: the effect of a structural change. Br J Gen Pract.

[B7] Olesen F, Jolleys JV (1994). Out of hours service: the Danish solution examined. BMJ.

[B8] Salisbury C (1997). Evaluation of a general practice out of hours cooperative: a questionnaire survey of general practitioners. BMJ.

[B9] Giesen P, Franssen E, Mokkink H, Bosch W van den, van Vugt A, Grol R (2006). Patients either contacting a general practice cooperative or accident and emergency department out of hours: a comparison. Emerg Med J.

[B10] van Duijn NP, Weert HCPM, van Scholte D, Bindels PJE (1998). Out-of-hours: primary care clinic or hospital emergency department?. Eur J Gen Pract.

[B11] van Uden CJ, Winkens RA, Wesseling GJ, Crebolder HF, van Schayck CP (2003). Use of out of hours services: a comparison between two organisations. Emerg Med J.

[B12] Hutchison B, Ostbye T, Barnsley J, Stewart M, Mathews M, Campbell MK (2003). Patient satisfaction and quality of care in walk-in clinics, family practices and emergency departments: the Ontario Walk-In Clinic Study. CMAJ.

[B13] Leibowitz R, Day S, Dunt D (2003). A systematic review of the effect of different models of after-hours primary medical care services on clinical outcome, medical workload, and patient and GP satisfaction. Fam Pract.

[B14] van Uden CJ, Ament AJ, Voss GB, Wesseling G, Winkens RA, van Schayck OC (2006). Out-of-hours primary care. Implications of organisation on costs. BMC Fam Pract.

[B15] Health care systems in transition, World Health Organization Regional Office for Europe: country information. http://www.euro.who.int/countryinformation.

[B16] Starfield B, Shi L, Macinko J (2005). Contribution of primary care to health systems and health. Milbank Q.

[B17] Derkx HP, Rethans JJ, Muijtjens AM, Maiburg BH, Winkens R, van Rooij HG (2008). Quality of clinical aspects of call handling at Dutch out of hours centres: cross sectional national study. BMJ.

[B18] Giesen P, Ferwerda R, Tijssen R, Mokkink H, Drijver R, Bosch W van den (2007). Safety of telephone triage in general practitioner cooperatives: do triage nurses correctly estimate urgency?. Qual Saf Health Care.

[B19] Moriarty H, McLeod D, Dowell A (2003). Mystery shopping in health service evaluation. Br J Gen Pract.

[B20] Lee TJ, Baraff LJ, Guzy J, Johnson D, Woo H (2003). Does telephone triage delay significant medical treatment?: Advice nurse service vs on-call pediatricians. Arch Pediatr Adolesc Med.

[B21] Bunn F, Byrne G, Kendall S (2004). Telephone consultation and triage: effects on health care use and patient satisfaction. Cochrane Database Syst Rev.

[B22] Hallam L (1994). Primary medical care outside normal working hours: review of published work. BMJ.

[B23] Olesen F, Jolleys JV (1994). Out of hours service: the Danish solution examined. BMJ.

[B24] Salisbury C (1997). Evaluation of a general practice out of hours cooperative: a questionnaire survey of general practitioners. BMJ.

[B25] Christensen MB, Olesen F (1998). Out of hours service in Denmark: evaluation five years after reform. BMJ.

